# Reversal Nanoimprinted 3D Plasmonic Sensor Around Microposts for Cell and DNA Detection

**DOI:** 10.3390/bios16080443

**Published:** 2026-08-16

**Authors:** Yijun Cheng, Stella W. Pang

**Affiliations:** 1 Department of Electrical Engineering, City University of Hong Kong, Kowloon, Hong Kong, China; 2 Centre for Biosystems, Neuroscience, and Nanotechnology, City University of Hong Kong, Kowloon, Hong Kong, China; 3 State Key Laboratory of Terahertz and Millimeter Waves, City University of Hong Kong, Kowloon, Hong Kong, China

**Keywords:** 3D plasmonic sensor, reversal nanoimprint lithography, sidewall coverage, live cell sensing, DNA detection

## Abstract

Localized surface plasmon resonance biosensors are promising devices for label-free detection of live cells and biomolecules. However, typical plasmonic sensors have limited surface area, planar electromagnetic fields, and poor compatibility with three-dimensional (3D) interactions with cells or biomolecules. In this study, a 3D plasmonic sensor around microposts was developed using reversal nanoimprint lithography for highly sensitive cell and DNA detection. Au nanopillars were conformally integrated onto the bottom, sidewall, and top of microposts, forming additional sensing surface area along the sidewall of microposts for plasmonic sensing. The 3D plasmonic sensors exhibited tunable resonance peaks and refractive index (RI) sensitivities by varying the micropost height. The highest sensitivity of 1306 nm per RI unit was obtained from the sensor with 10 μm-tall microposts at a resonance wavelength of 1315 nm, which was significantly higher than that of typical planar plasmonic sensors. The platform was applied to live MC3T3-E1 cell detection, showing a resonance peak shift of 71 ± 11.6 nm at a cell concentration of 10^6^ cells/mL with a cell concentration ranging from 10^2^ to 10^6^ cells/mL. In addition, DNA hybridization detection was demonstrated over a concentration range of 10^−15^–10^−7^ M complementary target DNA, with a resonance shift of 68 ± 2.5 nm observed at 10^−7^ M target DNA concentration. The 3D plasmonic sensor provides a scalable device for additional plasmonic biointerfaces with enhanced analyte accessibility and light–matter interactions. This platform offers high-sensitivity biosensing involving live cells, nucleic acids, and other biological targets.

## 1. Introduction

Live cell analysis and DNA detection play indispensable roles in biomedical research, disease diagnosis, drug discovery, and personalized medicine [1,2,3,4]. To date, numerous analytical approaches, including fluorescence assays, polymerase chain reaction (PCR), enzyme-linked immunosorbent assays, electrochemical biosensors, and surface-enhanced Raman scattering (SERS), have been extensively explored for biological detection [5,6]. Fluorescence-based techniques offer high specificity and sensitivity but generally require fluorescent labeling, complicated sample preparation, and expensive optical instrumentation, while also suffering from photobleaching and phototoxicity during measurements [7]. PCR-based methods enable highly sensitive nucleic acid analysis; however, they rely on complex thermal cycling, amplification procedures, and sophisticated equipment [8]. Electrochemical biosensors and SERS platforms have been demonstrated to provide biomolecular detection, yet they frequently face challenges associated with limited sensing areas, signal reproducibility, unstable hotspot distributions, and difficulties in quantitative analysis [9,10]. These limitations have motivated the development of label-free, highly sensitive biosensing platforms for the detection of live cells and biomolecules.

Localized surface plasmon resonance (LSPR) biosensors have emerged as a promising platform for label-free biosensing owing to their high sensitivity, rapid optical response, and straightforward detection principles [11]. LSPR sensors exploit the collective oscillations of free electrons in metallic nanostructures to generate strongly localized electromagnetic fields that are extremely sensitive to refractive index (RI) variations in the immediate vicinity of the plasmonic sensors [12]. Consequently, cellular attachment and biomolecular recognition can be monitored through resonance peak shifts without the need for fluorescent or chemical labels [13]. Compared with conventional bioanalytical methods, LSPR biosensors offer several attractive features, including label-free detection, simplified sample preparation, miniaturization potential, and compatibility with multiplexed and high-throughput sensing platforms [14]. These advantages have made LSPR plasmonic sensors highly attractive for applications involving live-cell analysis and DNA hybridization detection [15].

Typical LSPR biosensors are predominantly based on two-dimensional (2D) nanostructures, including nanopillars, nanoholes, and nanodisks fabricated on planar substrates [16,17,18]. Although these architectures exhibit strong plasmonic responses and have demonstrated promising sensing capabilities, their sensing interfaces and electromagnetic fields are intrinsically confined to planar geometries. Consequently, the active sensing area and light–matter interaction volume are inherently restricted, limiting further improvements in sensing performance. These limitations become particularly significant for the detection of complex biological targets such as live cells and biomolecules. Live cells possess inherently three-dimensional (3D) morphologies and interact with their microenvironment through membrane protrusions and spatially distributed adhesion sites rather than a single planar interface [19,20]. Similarly, DNA hybridization and other biomolecular recognition processes involve volumetric diffusion and binding events that occur throughout the 3D space [21]. Therefore, typical planar plasmonic sensors often exhibit insufficient sensing surface area, confined electromagnetic interaction regions, and poor compatibility with intrinsically 3D biological interactions, highlighting the need for plasmonic sensors capable of supporting biosensing in the vertical orientation besides a 2D planar area.

To overcome these limitations, substantial efforts have been devoted to developing 3D plasmonic sensors that enhance light–matter interactions and expand active sensing interfaces beyond typical planar surfaces [22]. Various strategies, including multiple layer plasmonic structures, 3D photonic crystals, vertically stacked nanostructures, and porous metallic frameworks, have been explored to improve RI sensitivity by increasing surface area for sensing and areas with intensive electromagnetic fields [17,23,24]. Quasi-3D plasmon-enhanced infrared spectroscopy based on metamaterial absorbers integrated with dielectric nanopedestals has been reported, where the vertically distributed nanostructures enhanced localized electromagnetic fields and improved the light–matter interactions. Such quasi-3D architectures demonstrate that introducing out-of-plane structural elements can effectively modify the plasmonic coupling behavior and improve optical sensing performance [25,26]. Despite their promising performance, many of these approaches involve complex fabrication processes, such as multiple lithography steps, layer-by-layer assembly, precise alignment, or costly nanofabrication techniques, which limit scalability, reproducibility, and large-area manufacturing. More importantly, although these platforms are often referred to as 3D structures, plasmonic nanostructures in most reported designs remain primarily confined to bottom surfaces, while vertical and sidewall regions are either inaccessible or underutilized for sensing [27].

The difficulty of patterning nanoscale structures on non-planar sidewalls represents a major limitation of conventional nanofabrication technologies. Photolithography, electron-beam lithography, and nano-pattern transfer methods are primarily optimized for planar substrates and generally encounter substantial challenges when attempting to achieve nanopatterning over the vertical sidewalls [23,28,29]. Factors such as non-uniform exposure, insufficient resist coverage, shadowing effects during metal deposition, and poor pattern transfer fidelity severely hinder the formation of well-defined plasmonic nanostructures along vertical sidewalls. As a result, the sensing potential of sidewall regions has remained largely underexplored, despite their ability to substantially increase active sensing area, analyte accessibility, and light–matter interaction volume.

Reversal nanoimprint lithography offers a promising solution to overcome these limitations. Compared with conventional nanofabrication approaches, reversal nanoimprint enables scalable, low-cost, and high-throughput replication of nanoscale features with excellent reproducibility [30]. More importantly, this technique enables conformal transfer of nanopatterns onto non-planar and 3D surfaces that are difficult to access using traditional lithographic methods. By transferring flexible films with nanostructures onto pre-fabricated microstructures such as microposts, reversal nanoimprint allows direct integration of nanostructures over curved or vertical interfaces, providing unique opportunities for constructing plasmonic sensors with expanded sensing interfaces along the sidewall orientation. Despite these advantages, the application of reversal nanoimprint for fabricating 3D plasmonic biosensors with nanopatterned sidewalls remains largely unexplored.

Recently, 3D plasmonic platforms based on metallic nanoparticle-coated microneedles and microstructured arrays have attracted considerable interest for SERS applications due to their enlarged surface area and enhanced electromagnetic field intensity [31,32]. By coating Au nanoparticles onto microneedle arrays, significant Raman enhancement can be achieved through localized plasmonic coupling effects [33,34]. However, such approaches typically rely on randomly distributed metallic nanoparticles, resulting in non-uniform electromagnetic field distributions, limited reproducibility, and poor spectral controllability. Furthermore, most nanoparticle-coated microstructures are primarily optimized for SERS enhancement rather than tunable RI sensing, making controlled quantitative biosensing based on resonance peak changes challenging [35].

In this study, a reversal nanoimprinted 3D plasmonic sensor around micropost arrays was developed for highly sensitive detection of live cells and DNA. Au nanopillars were integrated not only on the bottom of the microposts, but also along the sidewalls and top of microposts, forming a 3D plasmonic sensing architecture with additional sensing area along the sidewalls. Compared with typical planar plasmonic sensors and nanoparticle-coated microstructures, this configuration substantially expands the effective sensing interface and promotes enhanced plasmonic interactions across multiple sensing regions, resulting in enhanced analyte accessibility and stronger light–matter interactions. The resonance characteristics and sensing performance were further tuned by varying the micropost height, achieving a maximum sensitivity of 1306 nm per refractive index unit (RIU) at a resonance wavelength of 1315 nm. The developed plasmonic sensor demonstrated label-free detection of live MC3T3-E1 cells over a concentration range of 10^2^ to 10^6^ cells/mL with a maximum resonance peak shift of 71 ± 11.6 nm, as well as ultrasensitive DNA hybridization detection from 10^−15^ to 10^−7^ M with a maximum peak shift of 68 ± 2.5 nm. These results demonstrate that reversal nanoimprint enables the extends conventional planar plasmonic sensing toward a 3D sensing interface with additional sensing areas, providing a scalable platform for advanced biosensing and biointerface engineering with high sensitivity.

## 2. Experiment and Methods

### 2.1. Fabrication Technology for 3D Plasmonic Sensor Around Microposts

**Figure 1:**
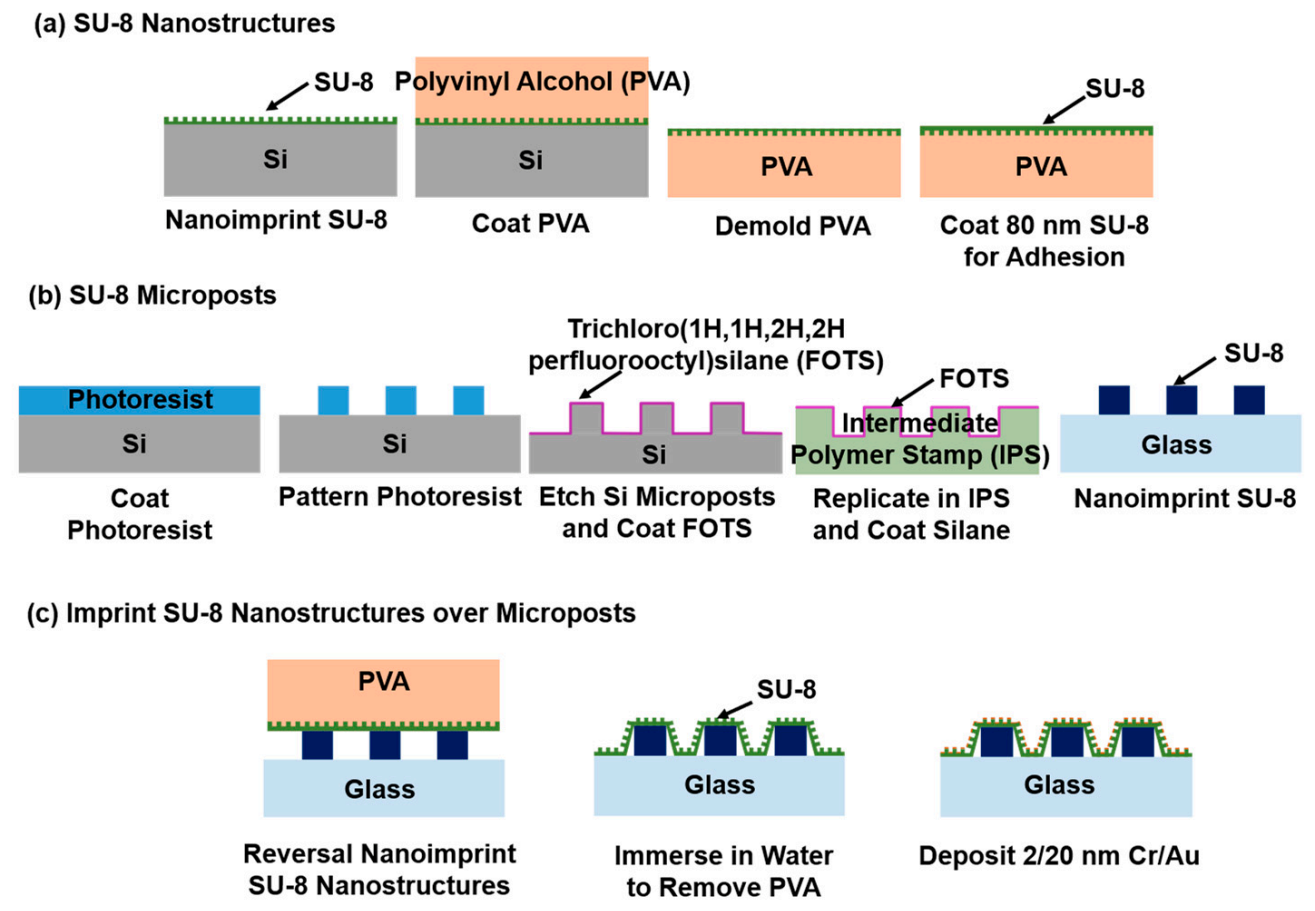
Schematic illustration of fabrication technology to reversal nanoimprint three-dimensional (3D) plasmonic sensor around micropost array. (**a**) SU-8 nanopillars (green) were first fabricated on Si substrate and transferred to flexible PVA stamp. (**b**) SU-8 microposts (blue) on glass were fabricated by photolithography, dry etching, and imprint with IPS on glass substrate. (**c**) Nanopillars were conformally transferred onto microposts through reversal nanoimprint, enabling nanopillars to form on bottom, sidewall, and top regions of microposts. After Cr/Au deposition, 3D plasmonic sensor was formed.

Figure 1a–c show the fabrication technology for conformally transferring nanopillars onto micropost arrays. First, the silicon (Si) substrate was sequentially cleaned with acetone, isopropanol, and deionized (DI) water for 10 min each, followed by baking at 105 °C for 10 min. To enhance surface hydrophilicity, the Si substrate was treated in an O_2_ plasma with 20 sccm O_2_, 100 mTorr pressure, and rf power of 100 W for 5 min. SU-8 photoresist (MicroChem, Westborough, MA, USA) was then spin-coated onto the Si substrate and baked at 65 and 95 °C for 2 min each. SU-8 nanopillars were subsequently fabricated by nanoimprint lithography using an intermediate polymer stamp (IPS), as illustrated in Figure 1a. The IPS was replicated from a nickel stamp under nanoimprint conditions of 150 °C and 40 bar for 5 min, followed by coating with trichloro(1H,1H,2H,2H-perfluorooctyl)silane to facilitate stamp release during nanoimprinting. Next, a 10% (weight by volume) polyvinyl alcohol (PVA) solution was cast onto the SU-8 nanopillar array. After curing at 25 °C for 3 days, a flexible PVA stamp containing the replicated SU-8 nanopillars was obtained.

Meanwhile, SU-8 micropost arrays were fabricated as illustrated in Figure 1b. Photoresist patterns were first defined on a Si substrate by UV photolithography, followed by development to form the micropost array pattern. A Si micropost stamp was subsequently fabricated by deep reactive ion etching using an etch condition of 138/11 sccm SF_6_/O_2_, 28 mTorr, 600 W coil rf power, and 14.8 W platen rf power at 13.56 MHz for 3 and 6 min to achieve 5 and 10 μm height, respectively. The Si micropost stamp was then replicated into an IPS, followed by a silane coating to facilitate demolding. Using the IPS, SU-8 micropost arrays were imprinted onto a glass substrate. Subsequently, the PVA stamp containing the SU-8 nanopillars was coated with a thin SU-8 layer with a thickness of 80 nm. Prior to reversal nanoimprinting, the flexible PVA transfer film carrying the SU-8 nanopillars was inverted such that the thin SU-8 layer faced the SU-8 micropost array. Owing to the flexibility of the PVA film, conformal contact was achieved over the bottom, sidewall, and top regions of the microposts by reversal nanoimprint, as shown in Figure 1c. The PVA film was nanoimprinted under the sequence of 45 °C, 10 bar, 2 s, no UV; 90 °C, 15 bar, 60 s, no UV; 90 °C, 15 bar, 20 s, UV on; 90 °C, 15 bar, 60 s, no UV. Following reversal nanoimprint, the sample was immersed in DI water to dissolve the PVA layer, resulting in conformal integration of nanopillars on the bottom, sidewall, and top regions of microposts. After dissolving the PVA layer in DI water, a plasma treatment with 20 sccm O_2_, 100 mTorr pressure, and rf power of 100 W for 45 s was performed to remove residual polymer film before Cr/Au deposition. This step improves surface cleanliness and metal adhesion while minimizing any influence on the replicated nanopillars. Finally, 2 nm-thick chromium (Cr) and 20 nm-thick gold (Au) were deposited by thermal evaporation to form the 3D plasmonic sensor around the micropost arrays.

**Figure 2:**
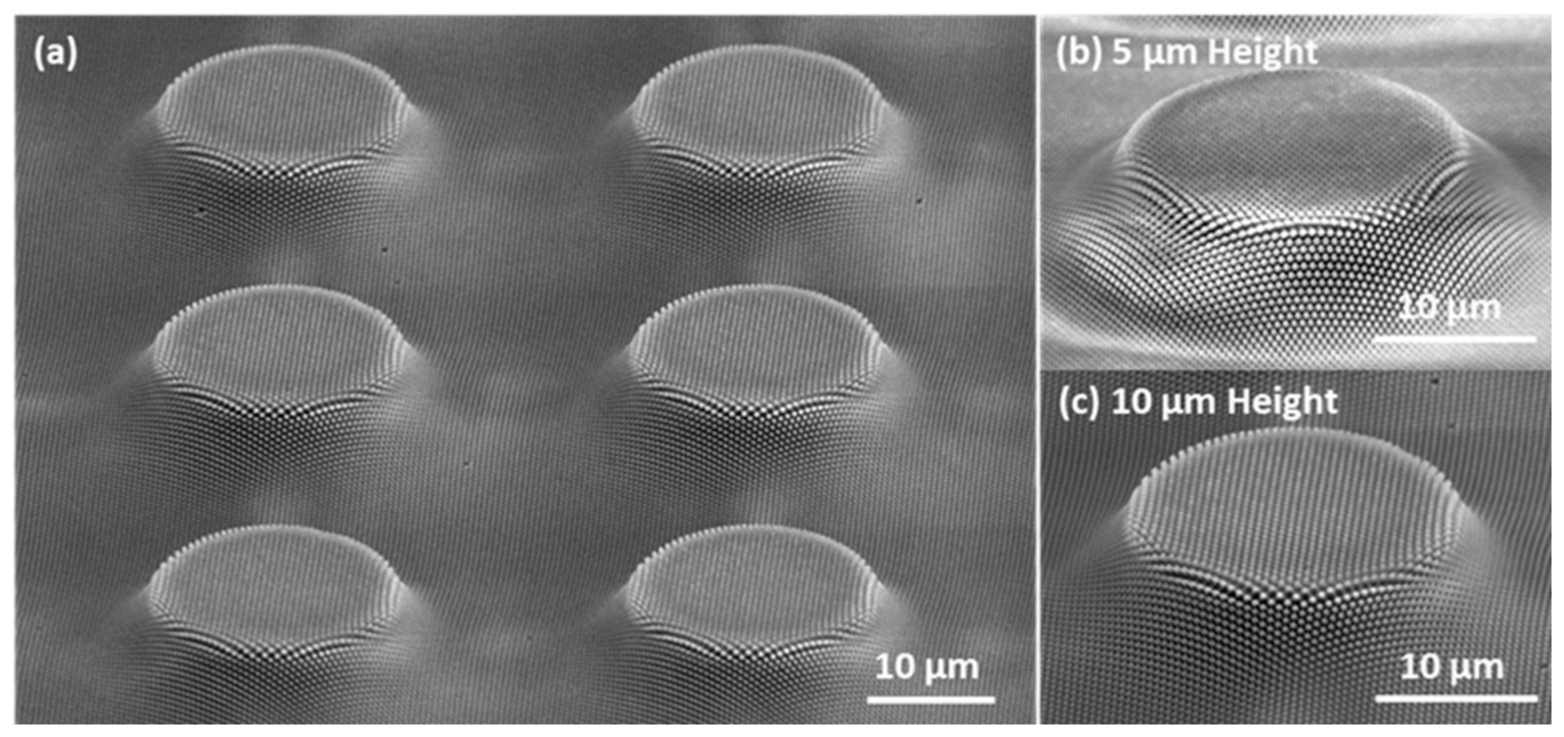
Three-dimensional plasmonic sensor around microposts. (**a**) Conformal integration of nanopillars on bottom, sidewall, and top regions of 10 μm-tall microposts. Enlarged images of 3D plasmonic sensor around microposts with heights of (**b**) 5 μm and (**c**) 10 μm.

The fabricated 3D plasmonic sensor around the micropost arrays had micropost diameters and spacing of 20 μm, with micropost heights of 5 or 10 μm, as shown in Figure 2. The nanopillars had diameters of 280 nm, spacing of 220 nm, and heights of 500 nm. The 3D plasmonic sensors around the 5 and 10 μm tall micropost arrays are shown in Figure 2. Similar conformal nanopillar coverage is observed for different micropost heights, indicating the robustness of the reversal nanoimprint for forming 3D plasmonic sensors with large sensing surfaces.

### 2.2. Measurement of Refractive Index Sensitivity

Optical characterization of the 3D plasmonic sensors was performed using a UV–visible–near infrared spectrophotometer (UV-3700, Shimadzu, Kyoto, Japan). A normal incident broadband light source with a wavelength range of 400–1600 nm was used to illuminate the 3D plasmonic sensor around the micropost arrays, and the extinction spectra were recorded. The RI sensitivity of the 3D plasmonic sensors was evaluated by immersing the sensors in certified RI liquids (Cargille Laboratories, Cedar Grove, NJ, USA) with refractive indices of 1.30, 1.33, and 1.36. The resonance peak shifts were measured as a function of RI variation to determine the sensing performance. For RI sensing measurements, a 10 μL droplet of certified RI liquid was directly dispensed onto the sensor surface to completely cover the sensing area. Transmission spectra were recorded immediately after liquid application. No coverslip was used during the measurements.

### 2.3. Cell Culture and Immobilization

An O_2_ plasma treatment was applied to the surface of the 3D plasmonic biosensors to enhance surface hydrophilicity and promote cell adhesion. This enabled MC3T3-E1 pre-osteoblastic cells (American type culture collection, CRL-2594, Manassas, VA, USA) to directly attach to the plasmonic sensor surface and be detected through optical measurements without requiring antibody functionalization. In addition, the 3D micropost further promoted cell attachment by providing increased surface contact area for cell–sensor interactions. Live MC3T3-E1 cells at different concentrations were suspended in 200 μL of Dulbecco’s Modified Eagle Medium (DMEM, Invitrogen, Carlsbad, CA, USA) and seeded onto the surface of the 3D plasmonic sensor in a culture dish. The cells were then incubated at 37 °C in a humidified atmosphere containing 5% CO_2_ for 6 h to allow cell attachment and immobilization on the hydrophilic sensor surface prior to optical measurements. MC3T3-E1 cells were selected as a representative adherent cell model because they exhibit robust attachment to platforms and pronounced cell membrane spreading and protrusion formation. These characteristics make them suitable for evaluating cellular interactions with the bottom, sidewall, and top sensing regions of the microposts. In addition, MC3T3-E1 cells are widely used in studies of cell–extracellular matrix interactions and bone disorders such as osteoporosis, providing a relevant model for assessing the potential of the proposed in vitro platform in 3D cell culture and monitoring. DMEM culture medium without cells was used as the blank control to establish the baseline resonance spectrum prior to measurements with cells. The resonance peak shifts reported throughout this paper were calculated relative to the corresponding DMEM baseline for each independently fabricated nanoplasmonic sensor, thereby eliminating variations arising from differences in the initial resonance peak positions.

### 2.4. Immobilization of DNA Probe and Hybridization with Complementary DNA Target

Prior to DNA functionalization, the 3D plasmonic sensors were washed three times with Milli-Q water and dried in air at 25 °C. After an O_2_ plasma treatment, 50 μL of 10^−6^ M thiol-modified DNA probe solution (5′-ACT CAT CAC CCT CCA AAC AAC AAT AAT CCT-SH-3′) (Integrated DNA Technologies, Singapore) prepared in DI water was added onto the surface of the 3D plasmonic sensor and incubated at 25 °C for 30 min. The thiol groups chemically bonded to the Au surface through Au–S interactions, forming a self-assembled monolayer of DNA probes on the sensor surface. The sensor was then rinsed with DI water to remove unbound DNA probes. Subsequently, 50 μL of fluorescein amidites(FAM)-labeled complementary target DNA solution (5′-AGG ATT ATT GTT GTT TGG AGG GTG ATG AGT AGT ATG TGG AGG TTA GGT GGA-FAM-3′) (Integrated DNA Technologies, Singapore) at different concentrations was introduced onto the sensor surface and incubated at 37 °C for 1 h to allow DNA hybridization. After hybridization, the sensor was washed with 1 mL of DI water to remove unbound target DNA and then air-dried prior to optical measurements. No concentration enrichment step was used during DNA hybridization detection. The molecular weights of the thiol-modified DNA probe and complementary target DNA were 9236 and 16,671 g/mol, respectively. To investigate the spatial distribution of the stained target DNA on the sidewalls of the 3D plasmonic nanopillars, confocal fluorescence z-stack imaging to scan from the top to the bottom of the 10 μm tall microposts was performed using a confocal inverted microscope (Stellaris 8, Leica, Wetzlar, Germany). The fluorescence signals from FAM-labeled target DNA were collected at successive focal planes along the z-axis. The acquired z-stack images were subsequently reconstructed into orthogonal cross-sectional views, enabling visualization of the vertical distribution of target DNA from the top to the bottom of the microposts.

### 2.5. Cell Imaging Using Scanning Electron Microscope

Following live-cell detection, the attached cells were fixed with 4% paraformaldehyde (Sigma-Aldrich, Burlington, MA, USA) for 15 min at room temperature. The samples were then washed sequentially with 1× phosphate-buffered saline (PBS, Sigma-Aldrich, Burlington, MA, USA) and 0.25× PBS for 5 min each, followed by rinsing twice with DI water for 10 min each. The cells were subsequently dehydrated through a graded ethanol series (30%, 50%, 70%, 80%, 95%, and 100%) for 5 min at each concentration. After dehydration, the samples were dried using a critical point dryer. A thin Au layer was then sputter-coated onto the sample surface using a thin film coater (Q150, Quorum Technologies Ltd., East Sussex, UK) to improve electrical conductivity for imaging. High-resolution images of the fixed cells were acquired using a field-emission scanning electron microscope (FE-SEM, SU5000, Hitachi, Tokyo, Japan) operated at an accelerating voltage of 10 kV. The high-resolution images were used to characterize cell morphology and cell–sensor interactions on the 3D plasmonic platforms.

## 3. Results and Discussion

### 3.1. Refractive Index Sensitivity of 3D Plasmonic Sensor Around Microposts

**Figure 3:**
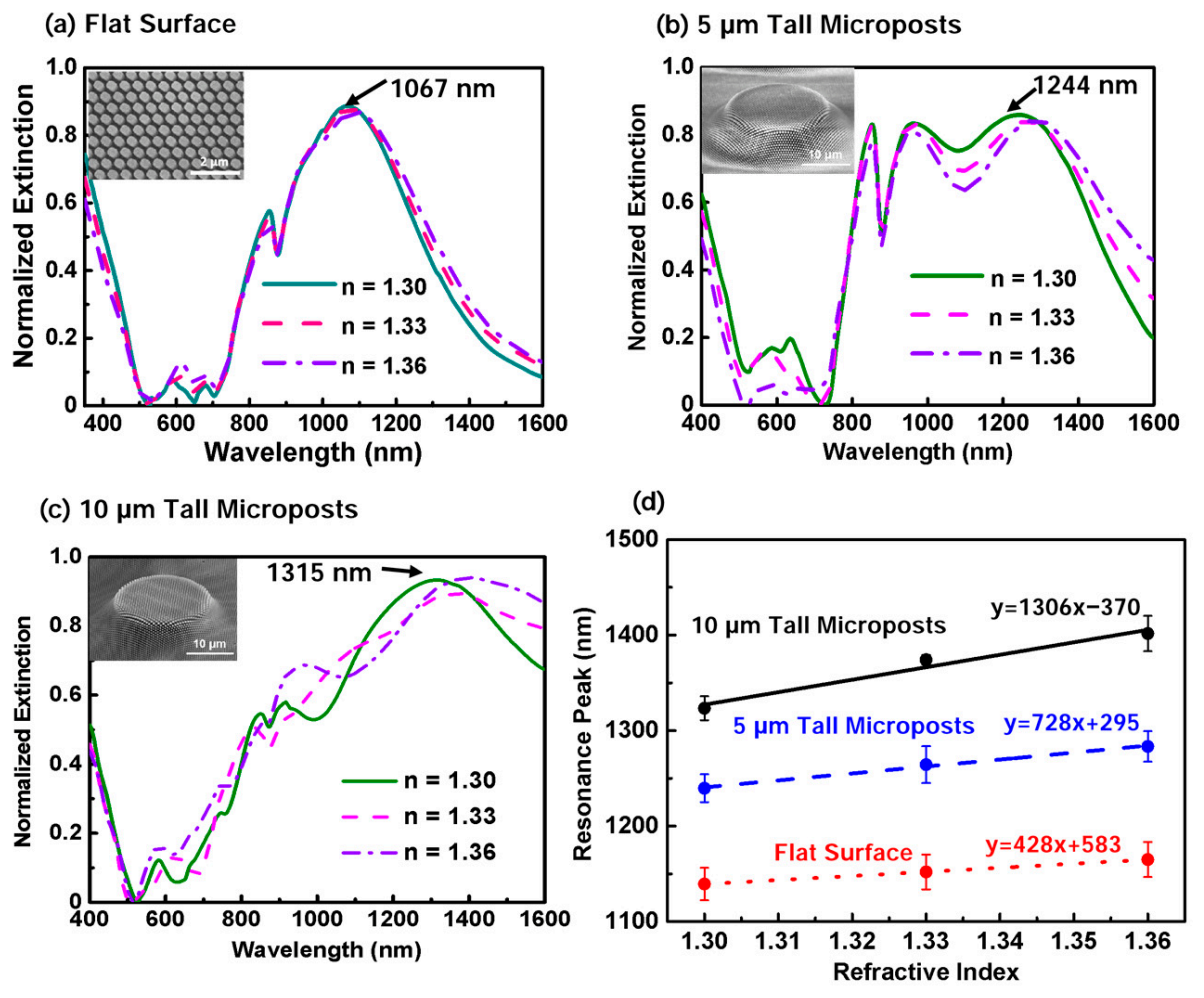
Normalized extinction and resonance peak shift as function of refractive index (RI) of certified RI liquids for Au nanopillars on (**a**) flat surface, (**b**) around 5 μm-tall microposts, and (**c**) around 10 μm-tall microposts. (**d**) Resonance peak shifts vs. RI for plasmonic sensors on flat surface, and around 5 and 10 μm-tall microposts. Error bars represent standard deviations of three independent measurements.

Figure 3 shows the normalized extinction spectra and resonance peak as a function of RI for Au nanopillars on a flat substrate (Figure 3a), around 5 μm-tall microposts (Figure 3b), and around 10 μm-tall microposts (Figure 3c). The insets in Figure 3a–c show micrographs of the corresponding nanostructures. The resonance characteristics and RI sensitivity of the nanopillar-based plasmonic platforms could be effectively tuned by varying the micropost height. Certified RI liquids with RI ranging from 1.30 to 1.36 were used to characterize the optical responses of the sensors. As shown in Figure 3a, the Au nanopillars on the flat substrate exhibited a dominant resonance peak at 1067 nm. The resonance peak gradually red-shifted as the certified RI liquid increased from 1.30 to 1.36. The measured sensitivity of the resonance peak at 1067 nm was 428 nm/RIU, as shown in Figure 3d. After transferring the nanopillars onto the 5 μm-tall microposts, the dominant resonance peak shifted to 1244 nm, as shown in Figure 3b. Correspondingly, the sensitivity increased significantly to 728 nm/RIU, as shown in Figure 3d. Further increasing the micropost height to 10 μm resulted in additional redshifts of the resonance peaks, as shown in Figure 3c. The 3D plasmonic sensor around 10 μm-tall microposts exhibited a resonance peak at 1315 nm and achieved the highest sensitivity of 1306 nm/RIU, as shown in Figure 3d. These results indicate that increasing the micropost height effectively enhances plasmonic coupling and improves RI sensitivity, as shown in Supplementary Figure S1. The multiple resonance peaks observed from the 3D plasmonic structure are attributed to geometry-dependent and hybridized plasmonic modes supported by the Au nanopillars located on the bottom, sidewall, and top regions of the microposts. Although Au nanoparticles commonly exhibit localized surface plasmon resonances in the visible or short-wavelength near-infrared range, complex and strongly coupled Au nanoplasmonic structures can support longer-wavelength resonance peaks [12,17]. In the present design, coupling of the electromagnetic field among the laterally and vertically distributed plasmonic elements could produce multiple hybridized resonance modes, including the resonance peak near 1315 nm [36]. Periodic array additionally influences radiative coupling and the overall spectral line shape. Since the micropost dimension is substantially larger than the measured wavelength at 1315 nm, this peak is unlikely to originate solely from a conventional first-order diffractive lattice resonance. It is probably associated with the hybridized plasmonic mode of the Au nanopillars located on the bottom, sidewall, and top regions of the microposts.

The plasmonic sensor sensitivity increased from 428 nm/RIU for flat surface to 1306 nm/RIU for nanopillars formed on 10 μm-tall microposts. Redshifts of the resonance peaks were observed with increasing micropost height. The redshift observed with increasing micropost height is attributed primarily to height-dependent redistribution and hybridization of the plasmonic modes supported by the top, sidewall, and bottom Au-coated nanopillars. As the nanopillar dimension, metal thickness, and constituent materials remained unchanged, increasing the micropost height extends the vertical plasmonic region along the sidewalls and hence modifies the relative modal contributions and electromagnetic field coupling among the nanopillars in the vertical regions. This promotes the formation of a hybridized mode and shifts the corresponding resonance peak toward longer wavelengths. Moreover, vertical phase-retardation, multiple-scattering, or cavity-like effects may also contribute to the peak shifts. The redshift of resonance peaks with increasing micropost height further suggests that the vertically extended nanopillar architecture alters the effective optical environment surrounding the plasmonic nanostructures. These results demonstrate that micropost height provides an effective parameter for tuning resonance characteristics and optimizing the sensing performance of the proposed nanopillar-based plasmonic platform [37,38].

Previous quasi-3D, multilayered, and 3D plasmonic architectures have demonstrated that vertically distributed metallic nanostructures can support multiple hybridized optical modes over a broad spectral range extending from the visible to the near-infrared, depending on the structural geometry and coupling mechanism [12,14,39]. However, the maximum sensitivities reported for these structures typically ranged from 230 to 496 nm/RIU. In contrast, the present 3D plasmonic sensor achieved a significantly higher sensitivity up to 1306 nm/RIU, representing a substantial improvement over previously reported plasmonic sensors. The enhanced RI sensitivity of the 3D plasmonic sensor is not solely attributed to the enlarged physical surface area provided by the sidewalls. Instead, the improvement originates from the expanded 3D active sensing region and the enhanced interaction between the localized electromagnetic fields and the surrounding microenvironment. The distribution of the Au nanopillars along the bottom, sidewall, and top regions of the microposts increases the accessible sensing volume and allows analytes to interact with multiple Au plasmonic nanopillars, thereby improving the effective overlap between the sensing environment and the plasmonic modes. Similar enhancement mechanisms have been reported in other 3D plasmonic architectures, where additional metallic nanostructures improve sensing performance by extending the electromagnetic field interaction region beyond conventional planar configurations [12,14,17]. In the quasi-3D nanosquare and 3D multilayered plasmonic biosensors, the localized electromagnetic fields extended around vertically distributed metallic nanostructures and multiple sensing interfaces, enhancing the interactions between analytes and plasmonic modes. The ultrahigh sensitivity, together with multiple resonance modes in the visible and near-infrared regions, makes the proposed platform highly promising for sensitive detection of live cells and DNAs. In addition, the gradual increase in resonance peak width with increasing RI could be originated from the modified dielectric loading and changes in the damping characteristics of the coupled plasmonic modes. In general, the width of plasmonic resonance peak is governed by the combined contributions of radiative damping, intrinsic metal loss, and mode coupling [17,40]. For nanopillars around 10 μm-tall microposts, the FWHM increased from 161 to 228 nm as RI of the medium changed from 1.30 to 1.36, as shown in Supplementary Figure S2.

### 3.2. Live MC3T3-E1 Cell Detection

**Figure 4:**
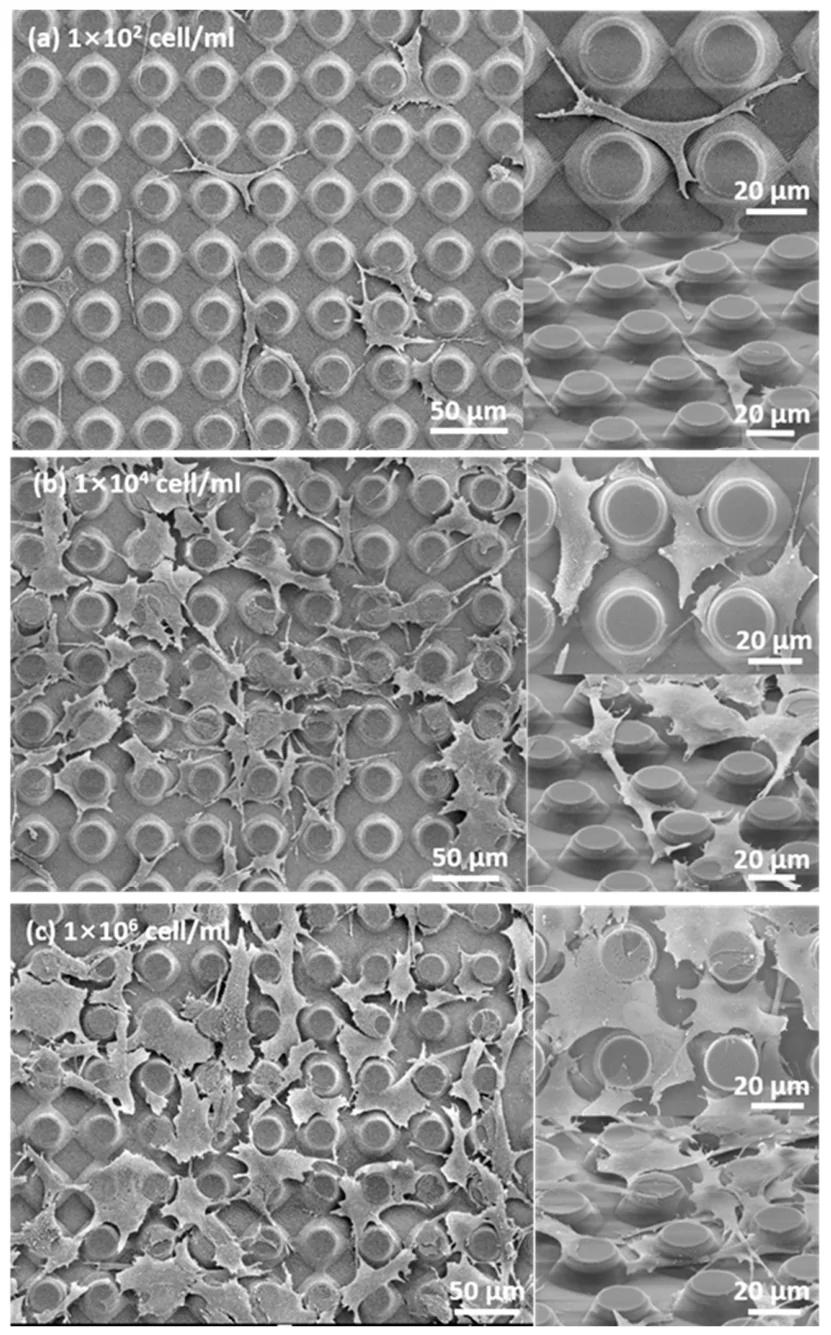
Micrographs of MC3T3-E1 cells on 3D plasmonic sensor around 10 μm-tall micropost arrays with cell concentrations of (**a**) 1 × 10^2^, (**b**) 1 × 10^4^, and (**c**) 1 × 10^6^ cell/mL. With increasing cell concentration, cell coverage on the sensor surface increased significantly. Enlarged top and tilted-view images reveal that cells interacted not only with top and bottom regions of microposts, but also with micropost sidewalls.

Since the plasmonic sensor based on 10 μm-tall microposts exhibited the highest sensitivity, all subsequent cell and DNA detections were performed using this sensor configuration. Figure 4 shows micrographs of MC3T3-E1 cells immobilized on the 3D plasmonic sensor around 10 μm tall microposts with cell concentrations ranging from 10^2^ to 10^6^ cells/mL. At the lowest cell concentration of 10^2^ cells/mL, MC3T3-E1 cells were sparsely distributed across the sensor surface, as shown in Figure 4a. Most cells were located in the bottom regions between microposts. However, cellular extensions also interacted with the sidewall regions of the microposts, providing additional contact areas between cells and the plasmonic sensing interface.

At a higher cell concentration of 10^4^ cells/mL, as shown in Figure 4b, the cell coverage increased significantly. Cells interacted not only with the bottom regions but also with the sidewalls and top surfaces of the microposts, resulting in substantially increased contact area between the cells and the plasmonic sensor. The enhanced cell coverage altered the local microenvironment surrounding the plasmonic sensor, leading to a measurable redshift in the resonance peak. When the cell concentration further increased to 10^6^ cells/mL, extensive cell coverage was observed throughout the micropost array, as shown in Figure 4c. From the top-view images, MC3T3-E1 cells had extensive contact with the 3D plasmonic sensor on the bottom, sidewall, and top of the microposts. From the tilted-view images, some cell protrusions extended on top of other cells and contacted plasmonic sensors on sidewalls of multiple microposts. Compared with conventional planar plasmonic sensors, where the sensing interface is mainly confined to a single surface, the proposed architecture provides plasmonic sensing regions distributed across different surfaces of the micropost structures. As a result, even under high cell-density conditions with cells overlaid on top of other cells, the cell membrane and protrusions can still be detected by the sidewall plasmonic sensor of the micropost array. This multi-surface sensing configuration enables sensitive live-cell detection over a broad concentration range.

**Figure 5:**
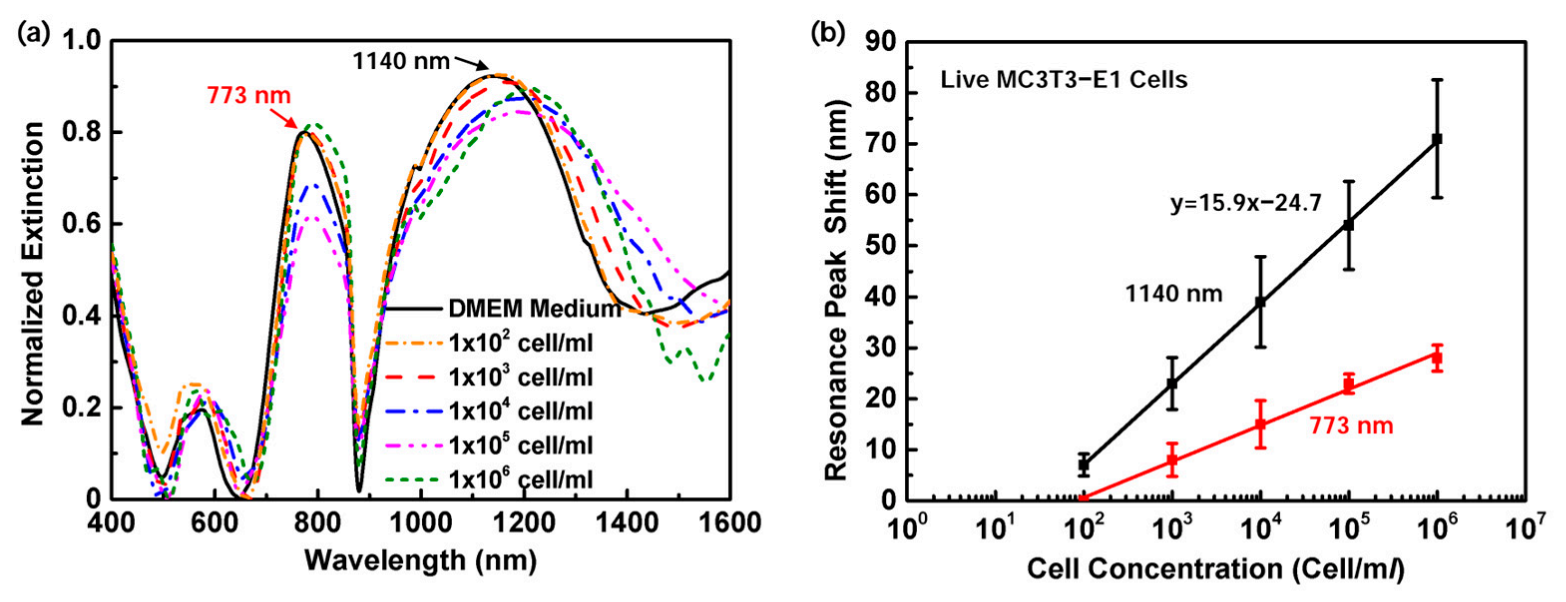
Optical characterization of live-cell detection using 3D plasmonic sensor around 10 μm tall microposts. (**a**) Normalized extinction spectra of plasmonic sensor in DMEM medium with MC3T3-E1 cells at different concentrations, resulting in concentration-dependent redshifts of resonance peaks. (**b**) Resonance peak shift as function of MC3T3-E1 cell concentration, showing live cell concentration detection down to 10^2^ cell/mL. Error bars represent the standard deviation of three independent measurements.

Figure 5 shows the optical responses of the 3D plasmonic sensor around 10 μm-tall microposts for live MC3T3-E1 cell detection at different cell concentrations. As shown in Figure 5a, two dominant resonance peaks at 773 and 1140 nm were observed with live cells in the DMEM medium. Both resonance peaks exhibited redshifts after live MC3T3-E1 cells at different concentrations were seeded onto the sensor surface, indicating concentration-dependent changes in the local RI surrounding. In this study, SU-8 nanopillars had a diameter of 280 nm, spacing of 220 nm., and 500 nm tall. Although the overall size of MC3T3-E1 cells, in the range of 15 to 30 μm, was significantly larger than the nanopillar dimensions, nanoscale cellular protrusions, including microvilli and filopodia, could interact and contact the nanopillar arrays directly, as shown in Supplementary Figure S3. Filopodia generally exhibit diameters below 300 nm, while microvilli typically have diameters of 100 nm [41,42]. The micrographs show that the cell membrane and membrane protrusions extended across the nanopillar-covered bottom, sidewall, and top regions of the microposts, fully utilizing the 3D sensing interfaces of the nanoplasmonics sensor. Accordingly, the measured resonance peak shifts are attributed to the changes in the effective local refractive index caused by cell attachment and spreading of the extensions from the cell membrane over the plasmonic sensing regions.

More importantly, unlike typical planar plasmonic sensors, the proposed plasmonic sensor investigated in this study contains active nanopillars sensing regions not only on the bottom surfaces, but also along the sidewalls and top of the microposts. This configuration enables cell sensing with large surface contact area, allowing cells to interact with plasmonic sensors throughout multiple regions of the micropost array. As cell concentration increased, more cells attached to the bottom, sidewall, and top of the micropost array, resulting in a larger effective sensing volume and stronger light–matter interactions. For live cells at a high concentration of 10^6^ cells/mL, the resonance peaks at 773 and 1140 nm were redshifted by 28 ± 2.5 and 71 ± 11.6 nm to 801 ± 2.5 and 1211 ± 11.6 nm, respectively. The larger shift observed at the 1140 nm resonance peak suggests a higher sensitivity of the near-infrared resonance mode to cell-induced RI changes, likely due to deeper electromagnetic field penetration and enhanced interaction volume in local microenvironment [43]. Figure 5b shows that the detected resonance peak shifts as a function of live MC3T3-E1 cells concentration ranging from 10^2^ to 10^6^ cells/mL. The limit of detection (LOD) was determined using the 3σ criterion based on the blank response. The resonance wavelength of the blank DMEM medium was measured 10 times repeatedly under identical experimental conditions, and the standard deviation σ of the blank response was calculated. The calibration curve between the resonance peak shift and the logarithm of the cell concentration was fitted as y = 15.9x − 24.7, where S, the slope of the fitted curve, is 15.9. The LOD was obtained using 3σblank/S, yielding a theoretical detection limit of approximately 56 cells/mL. The lowest experimentally validated concentration in this study was 100 cells/mL. The large resonance shifts observed over this concentration range demonstrate the high sensitivity of the proposed platform for live-cell detection. Compared with conventional imaging-based techniques such as confocal microscopy, which are often expensive, time-consuming, and require complex instrumentation, the fabricated plasmonic sensor platform shown in this work enables label-free detection of live cells in culture environments. These capabilities make the sensor platform particularly attractive for live-cell monitoring, cell migration study, and dynamic analysis of cellular responses to microenvironmental changes and therapeutic treatments [3].

### 3.3. Resonance Peak Shift as Function of Complementary DNA Target Concentration

**Figure 6:**
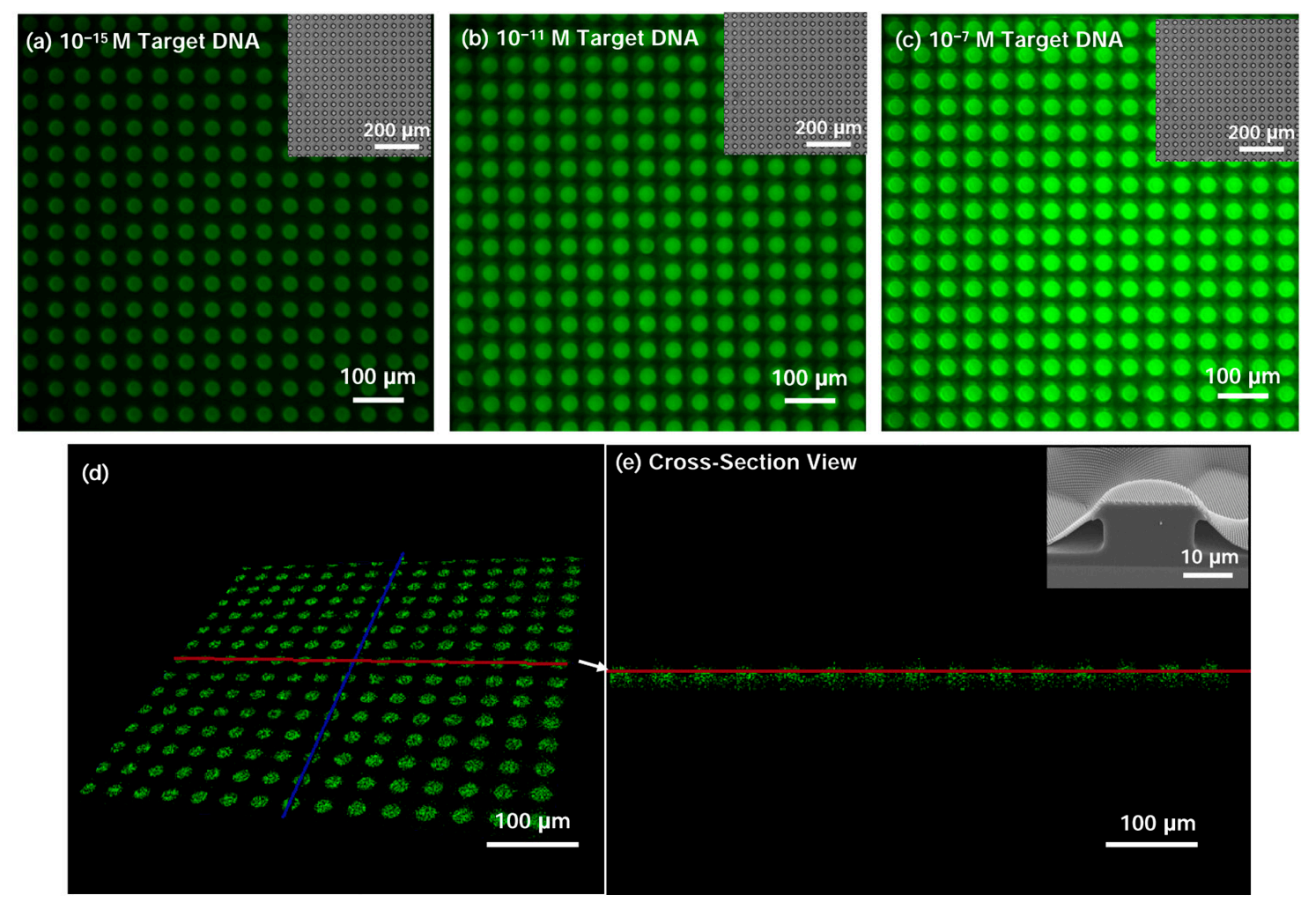
Fluorescence signal of DNA detected by 3D plasmonic sensor around 10 μm-tall microposts at concentrations of (**a**) 10^−15^, (**b**) 10^−11^, and (**c**) 10^−7^ M. (**d**,**e**) Fluorescence signal of target DNA detected by 3D plasmonic sensor along sidewalls of 10 μm tall microposts. Inset in (**e**) shows nanoplasmonic sensor around micropost.

Figure 6 shows the fluorescence characterization of DNA hybridization on the reversal nanoimprinted plasmonic sensor based on the 10 μm tall micropost arrays. Thiol-modified probe DNA was first immobilized onto the Au-coated plasmonic surface through Au–S bonding, followed by the introduction of fluorescently labeled complementary target DNA for hybridization analysis. Fluorescence images obtained at target DNA concentrations of 10^−15^, 10^−11^, and 10^−7^ M are shown in Figure 6a–c, respectively. The results clearly demonstrate successful and selective hybridization between the immobilized probe DNA and complementary target DNA on the 3D plasmonic sensor. Fluorescence signals were observed after hybridization, confirming effective target DNA capture on the sensor surface. Furthermore, the fluorescence intensity increased with increasing target DNA concentration, indicating concentration-dependent DNA binding behavior. The enhanced fluorescence intensity at higher concentrations suggests increased occupancy of target DNA molecules across the plasmonic sensing regions. These results confirm successful surface functionalization of the 3D plasmonic sensor and demonstrate its capability for efficient biomolecular capture over a wide concentration range.

To further investigate the spatial distribution of DNA binding on the plasmonic sensor around the microposts, confocal fluorescence microscopy was used to analyze the distribution of the target DNA, as shown in Figure 6d,e. The reconstructed fluorescence images revealed that the stained target DNA was attached not only to the probe DNA on the plasmonic sensor on the top of the microposts, but also along the sidewalls of the microposts. Cross-sectional views shown in Figure 6e revealed clearly fluorescence signals throughout the vertical direction of the microposts, indicating that the DNA molecules could effectively access and bind to the probe DNA located at different regions of the nanopillar-coated sensing interface. These results demonstrate that the micropost-based architecture provides additional accessible sensing regions compared with conventional planar plasmonic sensors, which may contribute to improved biomolecular interaction and detection sensitivity.

**Figure 7:**
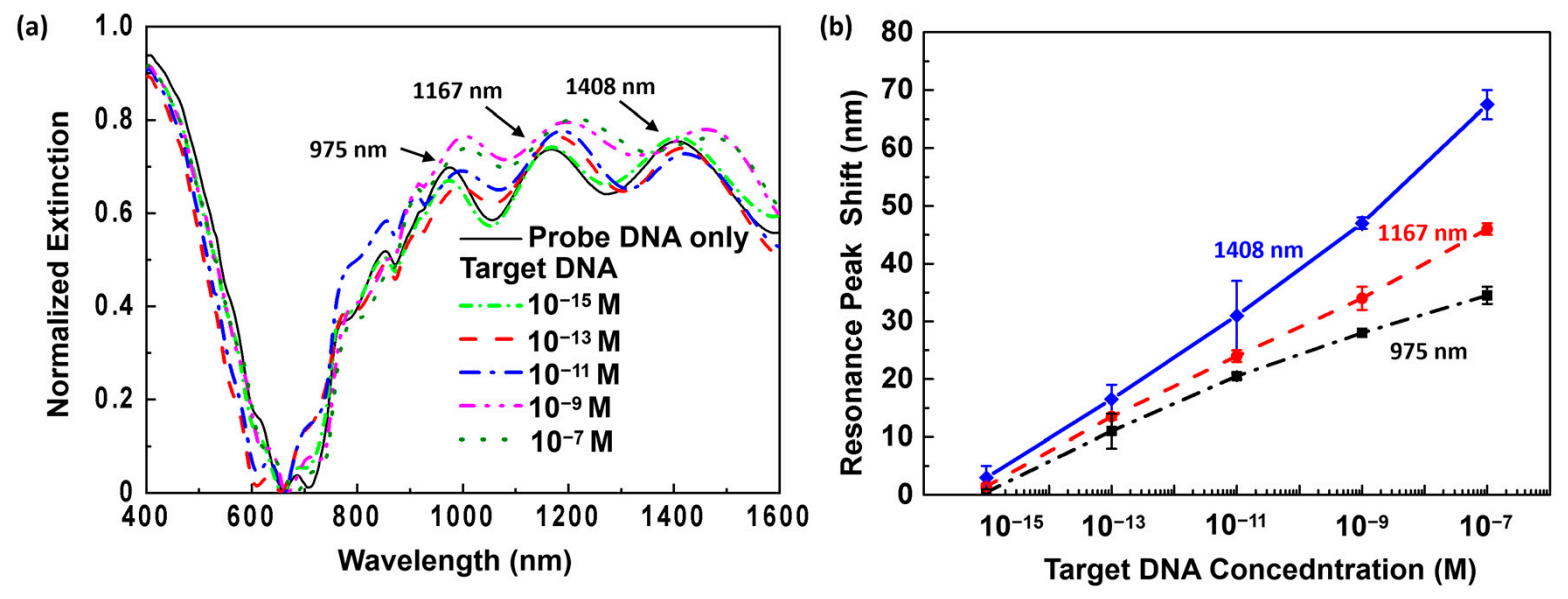
(**a**) Normalized extinction spectra of 3D plasmonic sensor around 10 μm tall microposts with probe DNA and complementary target DNA with concentration ranging from 10^−15^ to 10^−7^ M. (**b**) Resonance peak shift as function of complementary target DNA concentration.

Figure 7 shows the optical responses of the nanopillar-coated micropost architecture for DNA hybridization detection. Figure 7a presents the normalized extinction spectra of the sensors after probe DNA immobilization, followed by hybridization with complementary target DNA with concentrations ranging from 10^−15^ to 10^−7^ M. All resonance peaks exhibited clear redshifts after hybridization with complementary target DNA, confirming successful DNA binding and concentration-dependent changes in the local RI surrounding the plasmonic nanostructures. As the concentration of complementary target DNA increased, larger resonance peak shifts were observed. Figure 7b shows that the resonance peak shifts increased with target DNA concentration over the range of 10^−15^ to 10^−7^ M. For higher target DNA concentrations of 10^−7^ M, larger resonance peak shifts were observed, with maximum peak shifts of 45 ± 1 and 68 ± 2.5 nm measured at resonance peaks of 1167 and 1408 nm, respectively. The enhanced resonance response can be attributed to the increased accessible sensing regions provided by nanopillars distributed on multiple surfaces of the micropost structures. Compared with conventional planar plasmonic sensors, this architecture allows biomolecular interactions to occur across a larger fraction of the plasmonic interface, resulting in a stronger perturbation of the local dielectric environment after DNA hybridization. To further evaluate sequence specificity, a non-complementary DNA sequence (5′-CAA AAA CAA AAA CAA AAA CAA AAA-3′) was examined as a negative control. With 10^−7^ M DNA concentration, only a small resonance peak shift of 4 nm ± 0.5 nm was observed for the non-complementary DNA sequence, whereas the complementary target DNA induced a pronounced redshift of 68 ± 2.5 nm. This confirmed that the measured optical response predominantly originated from sequence-specific DNA hybridization rather than nonspecific adsorption, as shown in Supplementary Figure S4. These results demonstrate that the proposed 3D plasmonic sensor enables highly sensitive and wide-range DNA detection from 10^−15^ to 10^−7^ M.

## 4. Conclusions

In summary, a 3D plasmonic sensor around microposts was successfully developed using reversal nanoimprint lithography, enabling conformal integration of Au nanopillars on the bottom, sidewall, and top regions of the microposts. This unique configuration significantly expands the active plasmonic sensing surfaces and enhances light–matter interactions compared with conventional 2D plasmonic sensors. The sensing performance of the developed 3D plasmonic sensor can be effectively tuned by varying the micropost height from 5 to 10 μm, leading to a substantial increase in RI sensitivity from 728 to 1306 nm/RIU. This improvement is attributed to the increased interactions with nanostructures along the sidewalls of the microposts. For live-cell detection, the sensor enabled label-free monitoring of MC3T3-E1 cells over a wide concentration range of 10^2^ to 10^6^ cells/mL, with a maximum resonance peak shift of 71 ± 11.6 nm at 10^6^ cell/mL. In addition, the fabricated plasmonic sensor resulted in highly sensitive DNA hybridization detection over a wide range of 10^−15^ to 10^−7^ M, with a maximum resonance peak shift of 68 ± 2.5 nm at 10^−7^ M. The demonstrated sensing ranges should be considered in the context of the intended applications. The live cell detection range of 10^2^–10^6^ cells/mL is suitable for in vitro cell-based studies, including monitoring of cell adhesion, proliferation, and interactions with engineered 3D microenvironments. The DNA detection range of 10^−15^–10^−7^ M reaches the femtomolar level and is comparable with reported plasmonic nucleic acid sensors. Although further validation in complex biological environment is required, these results demonstrate the potential of the proposed 3D plasmonic sensor as a versatile platform for label-free biosensing of low-concentration cells or biomolecules. A comparison with the state-of-the-art plasmonic biosensors is shown in Table 1 to highlight the sensing ranges and structural characteristics of different platforms. The 3D plasmonic sensor shown in this work achieved the highest sensitivity with cell concentration down to 10^2^ cells/mL and DNA concentration down to 10^−15^ M.

Although cultured cells and immobilized DNA were employed as representative proof-of-concept targets in this study, the proposed sensing platform is not limited to these applications. Owing to its conformal 3D plasmonic architecture, the sensor provides a substantially enlarged active sensing interface compared with conventional planar plasmonic sensors, allowing analytes to interact simultaneously with the sensing nanostructures on the bottom, sidewall, and top regions of the microposts. Such a configuration is particularly advantageous for applications involving complex 3D biological cells or biomolecules, where conventional planar sensors suffer from limited analyte accessibility. This 3D plasmonic sensor could be applied to label-free monitoring of cell adhesion, proliferation, migration, and differentiation in 3D scaffolds, organ-on-chip systems, and tissue engineering. Furthermore, the proposed platform can be integrated with microfluidic systems for highly sensitive detection of low-abundance biomolecules, such as nucleic acids, extracellular vesicles, proteins, bacteria, and viruses, by enhancing analyte transport and capturing efficiency within the 3D sensing interface. Therefore, the cell and DNA experiments presented in this work are proof-of-concept demonstrations validating the sensing capability of the proposed platform, while its broader significance lies in establishing a versatile 3D plasmonic sensor for label-free biosensing applications.

In addition, compared with conventional fluorescence-based assays, the proposed platform enables continuous monitoring of live cells without fluorescent labeling, thereby avoiding photobleaching, phototoxicity, and potential perturbation of normal cellular behaviors. The optical responses directly reflect changes in the local microenvironment at the sensor interface, enabling label-free evaluation of cell adhesion and morphology without staining or labeling. These features complement conventional optical imaging and demonstrate the potential of the proposed platform for studies of cell–surface interactions in 3D biological systems.

## Figures and Tables

**Table 1:** Comparison of representative plasmonic biosensors with 3D plasmonic sensor in this work.

Sensor Architecture	Target	Detection Range	Structural Feature
Au nanoparticle LSPR sensor [44]	DNA	0.3–10^3^ nM	Planar localized sensing surface
Quasi-3D plasmonic nanopillars [12]	Cells	5 × 10^2^–10^6^ cells/mL	Vertically coupled plasmonic structures
3D multilayered plasmonic sensor [14]	Cells/DNA	5 × 10^3^–5 × 10^5^ cells/mL 10^−14^–10^−7^ M	Multilayer plasmonic coupling
3D plasmonic photonic crystal sensor [17]	Exosomes	10^4^–10^11^ particles/mL	Plasmonic–photonic hybrid structure
This work	Cells/DNA	10^2^–10^6^ cells/mL 10^−15^–10^−7^ M	3D plasmonic sensor on bottom, sidewall, and top of microposts

## Data Availability

The original contributions presented in this study are included in the article/Supplementary Materials; further inquiries can be directed to the corresponding author.

## Supplementary Materials

The following supporting information can be downloaded at: https://www.mdpi.com/article/10.3390/bios16080443/s1, Figure S1: Increased plasmonic sensor sensitivity for nanopillars on flat surface, and around 5 and 10 μm tall microposts; Figure S2: Full width at half maximum (FWHM) of Resonance Peak for 3D plasmonic sensor around 10 μm tall microposts as function of refractive index: Figure S3: (a–d) Scanning electron micrographs of MC3T3-E1 cells on the 3D plasmonic sensor showing extensions from cell membrane including (a) filopodia, (b) microvilli, and (c-d) protrusions in close contact with Au nanopillars on bottom, sidewall, and top of microposts; Figure S4: Normalized extinction spectra of 3D plasmonic sensor after probe DNA immobilization, following incubation with 10^−7^ M non-complementary DNA sequence, and after hybridization with 10^−7^ M complementary target DNA.
