# Supplementary material for: Reversal Nanoimprinted 3D Plasmonic Sensor Around Microposts for Cell and DNA Detection
Source: Biosensors (Basel). 2026 Aug 16;16(8):443. doi: 10.3390/bios16080443 (PMC13510717; doi:10.3390/bios16080443)
Supplement: Supplementary file 1 [file biosensors-16-00443-s001.zip › biosensors-4450884-supplementary.pdf]

Electronic Supplementary Information (ESI)

Reversal Nanoimprinted 3D Plasmonic Sensor around Microposts for Cell and DNA Detection

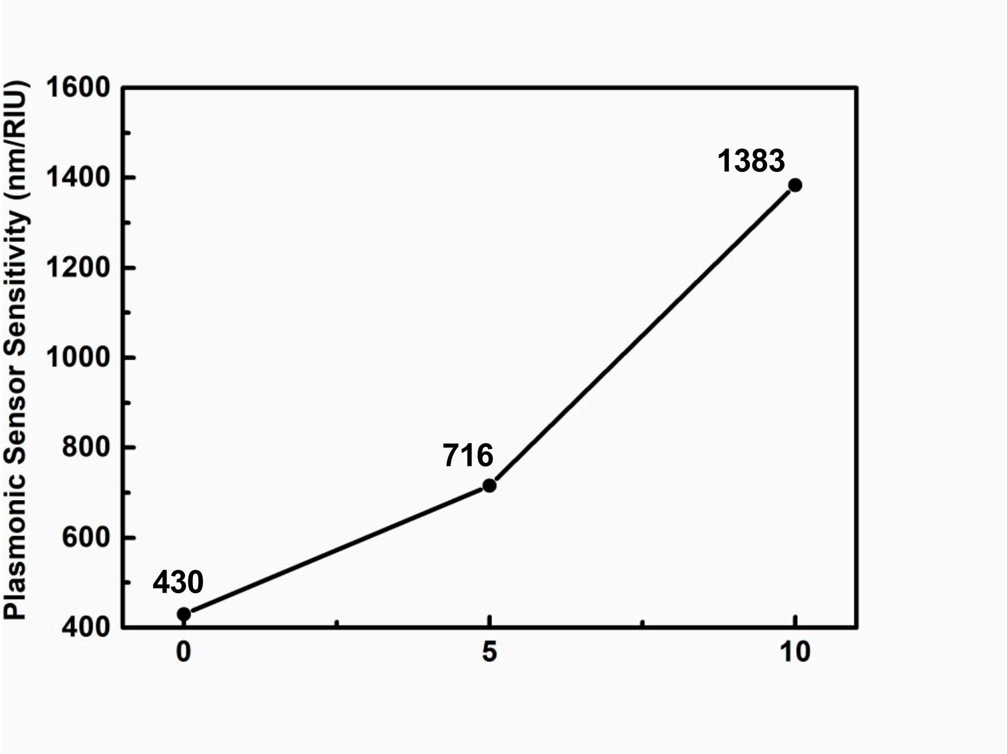

Supplementary Figure S1. Increased plasmonic sensor sensitivity for nanopillars on flat surface, and around 5 and 10 μm tall microposts.

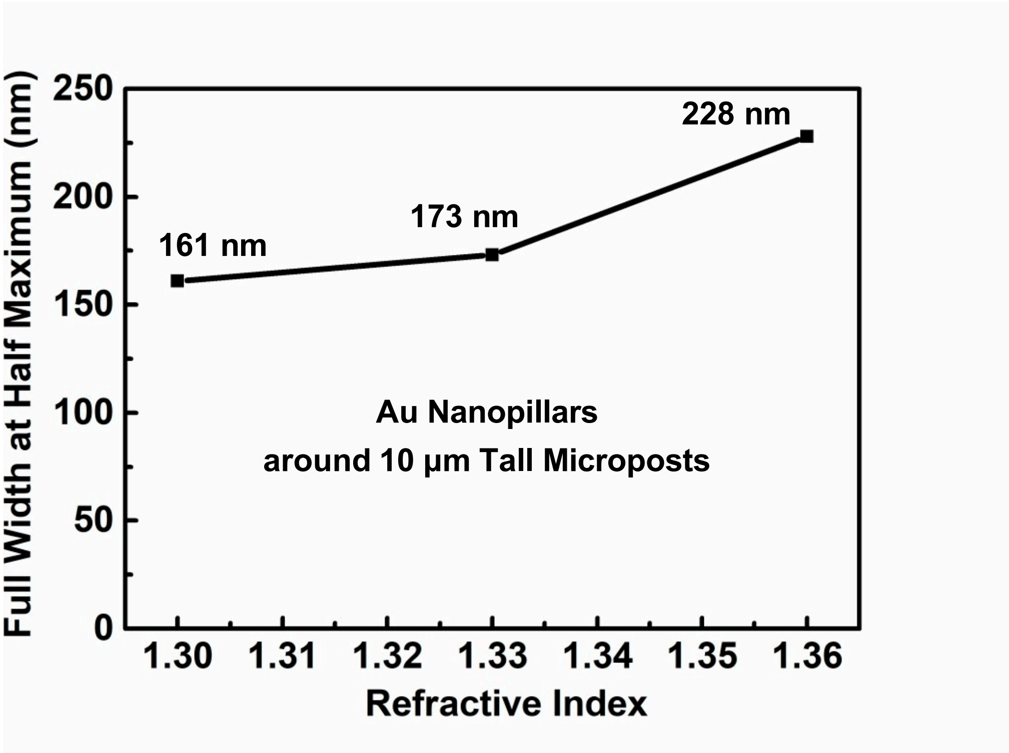

Supplementary Figure S2. Full width at half maximum (FWHM) of Resonance Peak for 3D plasmonic sensor around 10 μm tall microposts as function of refractive index.

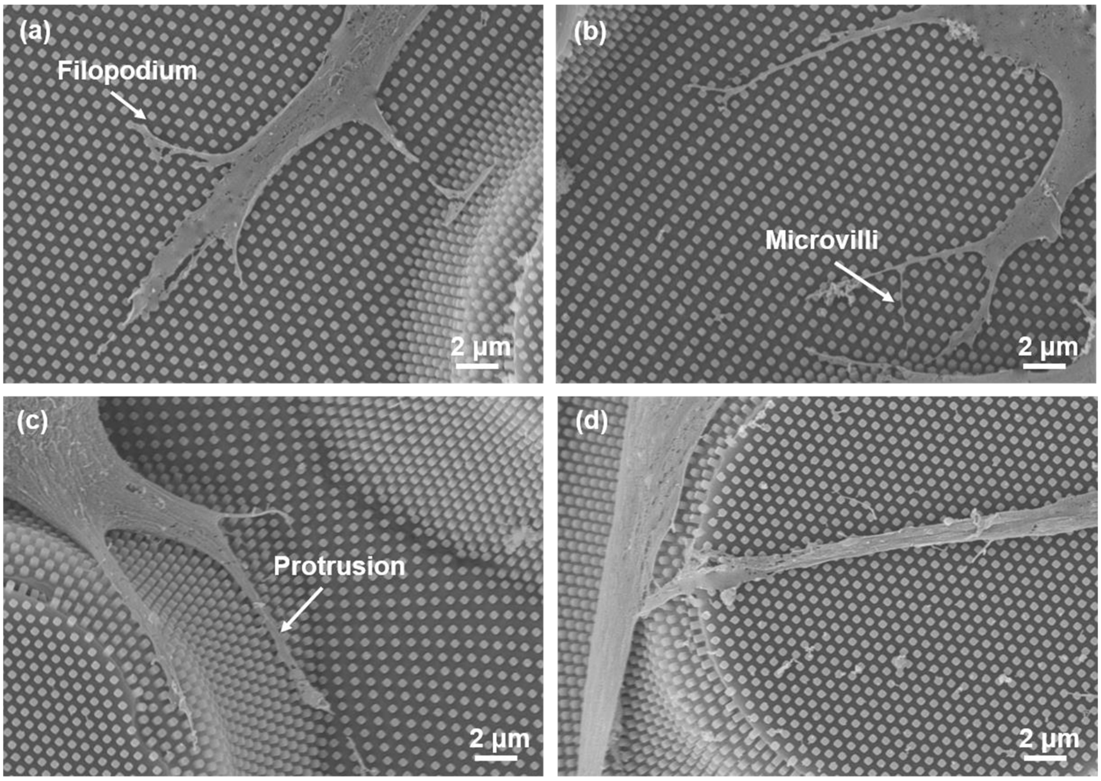

Supplementary Figure S3. (a–d) Scanning electron micrographs of MC3T3-E1 cells on the 3D plasmonic sensor showing extensions from cell membrane including (a) filopodia, (b) microvilli, and (c-d) protrusions in close contact with Au nanopillars on bottom, sidewall, and top of microposts.

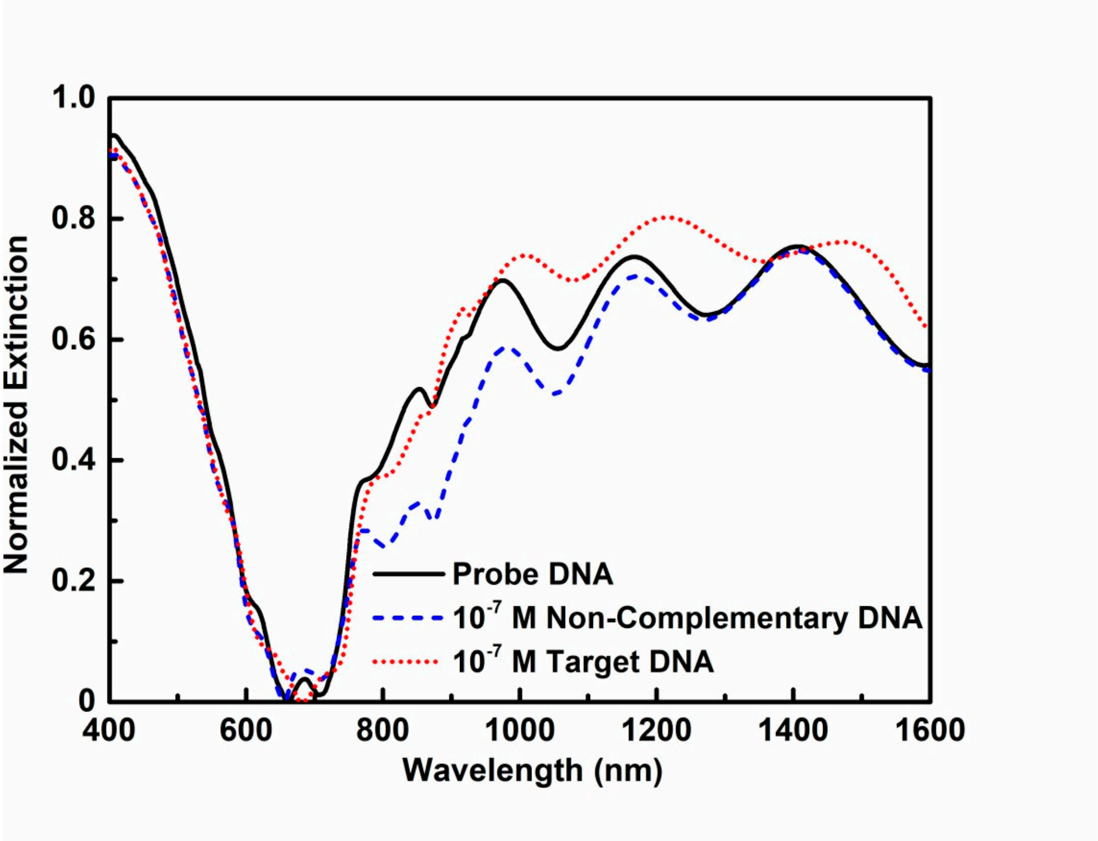

Supplementary Figure S4. Normalized extinction spectra of 3D plasmonic sensor after probe DNA immobilization, following incubation with 10^{-7} M non-complementary DNA sequence, and after hybridization with 10^{-7} M complementary target DNA.
